# Qualitative and psychometric evaluation of the PROMIS®-Fatigue SF-7a scale to assess fatigue in patients with moderately to severely active inflammatory bowel disease

**DOI:** 10.1186/s41687-023-00645-0

**Published:** 2023-11-14

**Authors:** Brian G. Feagan, William J. Sandborn, Bruce E. Sands, Yan Liu, Marion Vetter, Susan D. Mathias, Kuan-Hsiang Gary Huang, Jewel Johanns, Matthew Germinaro, Chenglong Han

**Affiliations:** 1 Western University and Alimentiv Inc, London, ON Canada; 2 University of California San Diego, La Jolla, CA USA; 3 Icahn School of Medicine at Mount Sinai, New York, NY USA; 4 Janssen Research & Development, LLC, Spring House, PA USA; 5 Health Outcomes Solutions, Winter Park, FL USA

**Keywords:** Patient-reported outcomes Measurement Information System® (PROMIS)-Fatigue Short Form 7a (SF-7a) scale, Crohn’s disease, Ulcerative colitis, Fatigue, Disease activity

## Abstract

**Background:**

This study evaluated the content validity and psychometric properties of the Patient-Reported Outcomes Measurement Information System® (PROMIS)-Fatigue Short Form 7a (SF-7a) v1.0 scale to determine its suitability in clinical trials to assess fatigue in patients with moderately to severely active Crohn’s disease (CD) and ulcerative colitis (UC).

**Methods:**

A qualitative interview assessed patients’ experience living with CD (N = 20) and UC (N = 19). The contents of the SF-7a scale were cognitively debriefed to evaluate content validity. A psychometric evaluation was performed using data from clinical trials of patients with CD (N = 360) and UC (N = 214). Correlations with Inflammatory Bowel Disease Questionnaire (IBDQ), Crohn’s Disease Activity Index (CDAI; CD only), and Mayo score (UC only) determined validity. The Patient Global Impression of Change (PGIC) was used to evaluate reliability and responsiveness to change. Using PGIC as an anchor, a preliminary threshold for clinically meaningful change was identified to define fatigue response in both CD and UC patients.

**Results:**

All patients reported fatigue as a common symptom. Patients confirmed SF-7a items were relevant to assessing fatigue, instructions and response options were clear, and its 7-day recall period was appropriate. Higher SF-7a scores were associated with higher disease activity (CDAI and Mayo score) and lower health-related quality of life (IBDQ), confirming known groups validity. The correlation of the SF-7a scale was higher with fatigue-related items. (r_s_ ≥ -0.70) than with items not directly associated with fatigue. Test-retest reliability was moderate to good (0.54–0.89) among patients with stable disease, and responsiveness to change in disease severity was demonstrated from baseline to Week 12. A ≥7point decrease was identified as a reasonable threshold to define clinically meaningful improvement.

**Conclusion:**

The SF-7a scale is a valid, reliable, and sensitive measure of fatigue in patients with moderately to severely active IBD and can be used to evaluate treatment response.

## Background

Inflammatory bowel disease (IBD) is a group of chronic relapsing disorders that includes Crohn’s disease (CD) and ulcerative colitis (UC) and is characterized by a progressive disease course that may include diarrhea, rectal bleeding, abdominal pain, bloody stool, and weight loss. IBD is debilitating and associated with a meaningful reduction in health-related quality of life (HRQoL), work impairment, and fatigue [1].

Fatigue is one of the most prevalent and burdensome symptoms among patients with IBD, with about 80% of patients with active disease reporting significant fatigue [1]. The Selecting Therapeutic Targets in Inflammatory Bowel Disease initiative of the International Organization for the Study of Inflammatory Bowel Diseases acknowledges that fatigue must be factored into the regular assessment of patients with IBD [2]. The pathogenesis of fatigue is complex and remains largely undefined; however, evidence suggests that it may involve physiological, psychological, and social aspects [1, 3]. For instance, chronic fatigue is more prevalent in patients with IBD and has been associated with pain intensity [4] and individual patients with IBD may have different fatigue experiences. This observation underscores the need to evaluate fatigue in patients with IBD. In addition, the content validity of a patient-reported outcome (PRO) instrument must be confirmed to effectively measure fatigue in clinical trials. However, clinical data related to fatigue in IBD patients remain limited.

The Patient-Reported Outcomes Measurement Information System® (PROMIS)-Fatigue Short Form 7a (SF-7a) scale evaluates the experience of fatigue and associated impact on daily life. The objectives of these analyses were to assess the content validity and evaluate the psychometric properties of the SF-7a scale in patients with moderately to severely active CD and UC.

## Methods

### Qualitative interview

Seven clinical sites in the United States (US) identified IBD patients to participate in qualitative interviews to assess the content validity of the SF-7a scale. Eligible patients were required to be adults ≥18 years of age with a confirmed diagnosis of moderately to severely active CD (defined as Crohn’s Disease Activity Index [CDAI] ≥220 and ≤450) and confirmed by radiography, histology, and/or endoscopy, or patients with confirmed diagnosis of moderately to severely active UC (defined as total Mayo score of 6 to 12 or a partial Mayo score of ≥5) for at least 3 months. Patients with a medical or psychiatric condition resulting in cognitive or any other impairment were excluded from the study.

Eligible patients were able to speak, read, and write in English and provided written informed consent prior to participating in the interview in which they were asked to complete a background questionnaire, which captured demographic and clinical information, as well as the Inflammatory Bowel Disease Questionnaire (IBDQ). A 1:1 combined-concept elicitation-cognitive debrief semi-structured interview was conducted via Zoom video conference (~ 60 min in duration). Patients were asked open-ended questions about living with IBD, symptoms they experience, and impacts on their lives using semi-structured interview guides (one for CD patients and one for UC patients) that were designed specifically for this study. Next, a cognitive debriefing interview was conducted in which patients were asked to provide feedback on their interpretation of (and any problems with) the instructions, each item of the SF-7a scale, response options, and recall period using questions, such as “In your own words, what is this question asking you to think about?; How did you respond to this question?; Why did you give that response?; Is this question clear or unclear? Why?”

This approach enabled an evaluation of the relevance, interpretability, clarity, and ease of understanding of all concepts and content of the SF-7a scale. All interviews were recorded and transcribed. Data from the interviews were coded using a qualitative data analysis software, MAXQDA (version 2020). Two coding dictionaries were developed (one for CD patients and one for UC patients) and used in the analysis of the transcripts. The codebook was used to organize and categorize concepts of interest from the interviews and included descriptions and examples for each code to ensure consistency across coders. Each transcript was coded by one coder, reviewed, summarized, and analyzed by a second coder who performed the analysis.

### Psychometric evaluation

#### Patient population

Data for the psychometric evaluation were available from participants enrolled in the GALAXI-1 (NCT03466411) and VEGA (NCT03662542) studies. GALAXI-1, a Phase 2, dose-ranging, double-blind, active- and placebo-controlled study, was designed to evaluate the efficacy and safety of guselkumab in patients with moderately to severely active CD. VEGA, a Phase 2a, randomized, double-blind, active-controlled, parallel-group, multicenter study, was designed to evaluate the efficacy and safety of combination induction therapy with guselkumab and golimumab in patients with moderately to severely active UC. Detailed descriptions of each study, including eligibility criteria, patient and disease characteristics, and key efficacy and safety outcomes have been presented elsewhere [5–11]. Here, we present results from the psychometric analysis using pooled data across treatment groups by each study.

### PROMIS-Fatigue SF-7a v1.0 scale

The SF-7a scale is a 7-item instrument that evaluates fatigue-related symptoms (i.e., tiredness, exhaustion, mental tiredness, and lack of energy) and associated impacts on daily activities (i.e., activity limitations related to work, self-care, and exercise) during the past 7 days with a 5-point Likert scale that ranges from “1 = Never” to “5 = Always” (Supplemental Table 1). Higher SF-7a scores indicate more fatigue. The raw score of SF-7a is converted into a standardized T-score based on the US national norm with a mean T-score of 50 and standard deviation (SD) of 10 [12]. Mean T-scores > 50 indicate the presence of fatigue.

### Anchor assessments for validation

The IBDQ is a 32-item IBD-specific questionnaire that has been adapted and validated into several languages and cultural milieus [13]. IBDQ is composed of 4 domains (bowel symptoms, systemic symptoms, emotional function, and social function) that are assessed within the past 14 days [14]. IBDQ items are scored on a 7-point Likert scale that ranges from “1 = Worst” to “7 = Best” yielding a total score that ranges from 32 to 224, with higher IBDQ total scores indicating better HRQoL. The Patient Global Impression of Change (PGIC) is a single item used to evaluate overall change in health status from the patient perspective using a 7-point Likert scale (“A lot better”, “Moderately better”, “A little better”, “Neither better, nor worse (no change)”, “A little worse”, “Moderately worse”, “A lot worse” [15].

The CDAI score, which is most commonly utilized to assess disease activity in patients with CD, ranges from 0 to 600, with higher CDAI scores indicating more severe active disease [16].

The Mayo score evaluates disease activity in UC patients and is comprised of 4 components: stool frequency, rectal bleeding, endoscopy, and physician’s global assessment [14]. Each is scored on a scale from 0 to 3, and the maximum total score is 12, with higher Mayo scores indicating more severe disease. The partial Mayo score uses 3 non-invasive components of the full Mayo score (i.e., stool frequency, rectal bleeding, and physician’s global assessment), with a maximum score of 9.

### Statistical analysis

Baseline demographic and clinical characteristics were summarized using descriptive statistics (mean ± SD), unless otherwise stated. Known groups validity assesses whether a measure can distinguish groups that are clinically different. Patients were stratified into 3 subgroups based on their HRQOL by IBDQ score [remission (no or mild impact): ≥170; moderate impact:169 − 110; severe impact: <110)] [17, 18], CD disease severity by CDAI score (remission: <150; mild: 150–219; moderate to severe: 220–450; very severe: >450) [19] or UC severity by partial Mayo score (remission: 0-1; mild: 2‐4; moderate: 5‐6; severe: >6). Mean SF-7a scores were summarized by defined subgroups and compared using analysis of variance.

Convergent validity evaluates whether measures that theoretically should be correlated are, in fact, observed to be highly correlated. Discriminant validity assesses whether measures that theoretically should not be correlated are, in fact, observed to be not correlated. The convergent and discriminant validity were evaluated by comparing Spearman’s correlation between the SF-7a scale and individual IBDQ items related to fatigue/energy level and items less likely related with fatigue.

Test-retest reliability is the process of reproducing identical measurement among stable patients. The intraclass correlation coefficients (ICC) of SF-7a were calculated using data from patients with stable disease defined by PGIC as “no change” from the baseline at Week 12. In general, ICC values below 0.50 indicate poor reliability, 0.50 to 0.75 moderate reliability, 0.75 to 0.90 good reliability, and > 0.90 excellent reliability [20].

Responsiveness, which measures how effective a scale detects change over time, was evaluated for the SF-7a scale by calculating the standardized effect size (SES), standardized response mean (SRM), and Guyatt’s statistic. The SES [21] was calculated as the difference in means between baseline and Week 12 in the SF-7a scale divided by the baseline SD. The SRM [22] was calculated as the difference in means between baseline and Week 12 divided by the SD of the change scores. The responsiveness statistic [23] was calculated as the difference in means between baseline and Week 12 in the SF-7a scale divided by the SD of the change in score for stable patients as assessed by the PGIC.

Using PGIC as an anchor variable, changes in the SF-7a scale were summarized across 7 response levels of PGIC. The clinically meaningful threshold was defined as the mean change in SF-7a scores among patients with PGIC response of “moderately better” (representing two levels of improvement) at Week 12 [24].

### Institutional review board

The study protocol for the qualitative research and informed consent forms were reviewed and approved by WCG Institutional Review Board prior to the start of the study on August 6, 2020.

## Results

### Qualitative interview

A total of 39 patients (20 CD and 19 UC) participated in the qualitative interview. Age and duration of disease were similar between CD and UC patients (Table 1). The majority of CD patients were male (60%) and the mean age was 43 years. Overall, the CD patient population had a mean disease duration of 14.9 years. The UC patient population was predominately female (68%) and the mean age was 46 years. Overall, UC patients had a mean disease duration of 11.1 years.

**Table 1:** Patient demographics and disease characteristics for CD and UC patients participating in the qualitative interview

	CD patients (N = 20)	UC patients (N = 19)
Age, years	43.1 (15.1)	46.1 (12.9)
Male, n (%)	12 (60)	6 (32)
Duration of disease, years	14.9 (9.6)	11.1 (9.0)

Data are being reported as mean (SD), unless otherwise specified. *CD*, Crohn’s disease; *UC*, ulcerative colitis

### Concept elicitation

All patients (100%) in both the CD and UC groups spontaneously reported fatigue as one of their most common symptoms (see patient quotes below). Other symptoms mentioned by patients included increased stool frequency (CD, 100%; UC, 100%), abdominal pain/cramping (CD, 95%; UC, 100%), diarrhea (CD, 100%; UC, 74%), urgency (CD, 80%; UC, 100%), joint pain (CD, 80%; UC, 11%), bloating (CD, 35%; UC, 37%), blood/mucus in stool (CD, 85%; UC, 100%), inability to pass stool despite urgency (CD, 15%; UC, 68%), rectal pain (CD, 10%; UC, 42%), rectal bleeding (CD, 60%; UC, 53%), gas (CD, 40%; UC, 16%), and nausea (CD, 25%; UC, 21%).

### Patient reflections on fatigue

Patients were given an opportunity to describe their fatigue based on their personal experiences. Patients reported that fatigue symptoms impacted their ability to do daily and physical activities. One CD patient said, “*For the most part, I’m pretty exhausted all the time. Of course, I have like the body of an 80-year-old, but yeah I mean I’m just wiped out all the time. I probably have to take a nap at least three or four days out of the week because I can’t make it through the full day without pain*.” Another CD patient noted, “*Yeah. I’ll wake up in the morning and I’ll be fine for a couple of hours, and all of a sudden out of nowhere you just feel drained and want to lay back down*.”

Similar quotes were provided from UC patients. One UC patient replied, “*Oh, lord, yes. You feel very, like you’re just destroyed. You know like you are just, there’s nothing left. You can’t, you’re just, like I want to lay down and just rest and you think if I can just rest then I’ll feel better and you don’t*.” Another UC patient mentioned, “*Slight fatigue on a daily basis, but if it’s inflamed most definitely. It’s very exhausting. It takes all the life out of me*.”

When describing fatigue experience, patients with CD or UC often talked about their fatigue intensity/severity and frequency together using expressions such as “constantly exhausted,” or “wiped out all the time”, indicating both fatigue frequency and intensity are meaningful attributes to evaluate fatigue experience.

### Cognitive debriefing

Cognitive debriefing of the PROMIS-Fatigue SF-7a revealed that the majority of CD and UC patients (> 75%) were able to accurately paraphrase all items, found that items were clear and easy to recall, relevant to fatigue, and easy to complete. Fatigue experience was easy to recall within the previous 7 days (Table 2).

**Table 2:** Cognitive debriefing of the PROMIS Fatigue SF-7a scale

	SF-7a scale	
	CD patients (N = 20)	UC patients (N = 19)
Accurately paraphrase?	90-100%^a^	90-100%^a^
Questions clear?	78-100%^b^	88-100%^b^
Easy to think about past 7 days?	95%^c^	100%
Questions relevant?	95%	79%
Questionnaire easy to complete?	95%	100%

*CD*, Crohn’s disease; *PROMIS*, Patient-Reported Outcomes Measurement Information System; *SF-7a*, Short Form-7a; *UC*, ulcerative colitis. ^a^The number of patients who were asked whether SF-7a questions were accurately paraphrased varied in CD patients from n = 9 to n = 15 and in UC patients from n = 7 to n = 15 for each SF-7a question. ^b^The number of patients who were asked whether SF-7a questions were clear varied in CD patients from n = 18 to n = 20 and in UC patients from n = 16 to n = 18 for each SF-7a question. ^c^Based on n = 19 patients

The cognitive debriefing allowed additional information to be gathered from the CD and UC patients based on the patient responses to the SF-7a. For example, 35% of CD patients indicated that work caused them to run out of energy, 64% noted that fatigue limited their job-related responsibilities, 31% reported being too tired to think clearly in the evening/at the end of the day, and 50% described strenuous exercise as lifting weights or doing crunches.

Similarly, 36% of UC patients indicated that daily activities/everything caused them to run out of energy, 60% noted that fatigue limited their work for pay responsibilities, 38% reported being too tired to think clearly at the end of the day, and 46% described strenuous exercise as weightlifting.

### Psychometric evaluation

Baseline demographics and clinical characteristics of patients with CD and UC are summarized in Table 3. Mean (SD) T-score values of PROMIS-Fatigue SF-7a at baseline were 59.0 (8.2) and 57.4 (8.4) for CD and UC patients, respectively, which were > 50 (the population norm) indicating fatigue.

**Table 3:** Patient and disease demographics at baseline in GALAXI (CD patients) and VEGA (UC patients)

	CD patients (N = 360)	UC patients (N = 214)
Age, years	39.3 (13.9)	38.4 (12.0)
Male, n (%)	217 (60.3)	116 (54.2)
Duration of disease, years	8.9 (8.8)	4.9 (5.0)
CDAI total score	305.2 (56.7)	
Mayo score		8.8 (1.4)
IBDQ total score	125.8 (33.3)^a^	116.5 (33.3)^b^
PROMIS Fatigue SF-7a score	59.0 (8.2)^a^	57.4 (8.4)^b^

CD and UC patients were derived from pooled treatment groups from GALAXI and VEGA studies, respectively. Data are being reported as mean (SD), unless otherwise specified. *CD*, Crohn’s disease; *CDAI*, Crohn’s Disease Activity Index; *IBDQ*, Inflammatory Bowel Disease Questionnaire; *PROMIS*, Patient-Reported Outcomes Measurement Information System; *SD*, standard deviation; *SF-7a*, Short Form-7a; *UC*, ulcerative colitis. ^a^Based on 353 CD patients. ^b^Based on 209 UC patients

### Known groups validity

**Fig. 1:**
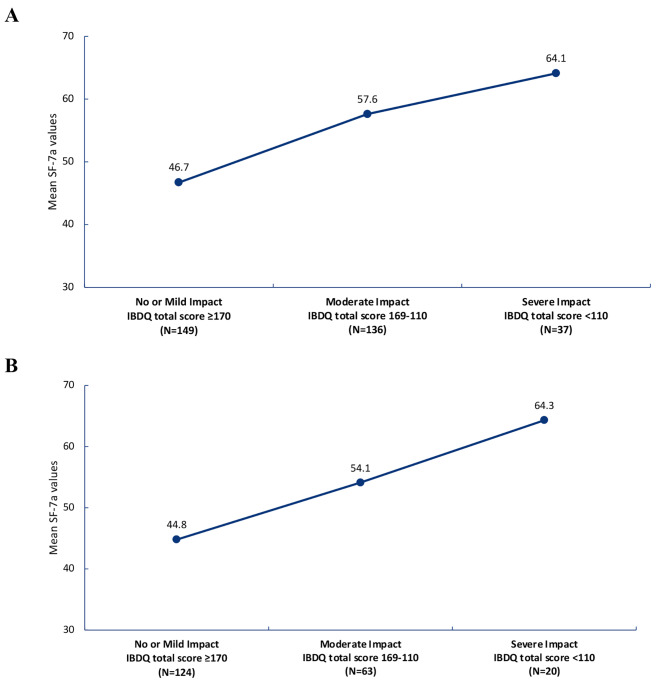
Mean PROMIS Fatigue SF-7a values, by impact on HRQOL by IBDQ total score at Week 12 in patients with (**A**) CD and (**B**) UC. *CD*, Crohn’s disease; *IBDQ*, Inflammatory Bowel Disease Questionnaire; *PROMIS*, Patient-Reported Outcomes Measurement Information System; *SF-7a*, Short Form-7a; *UC*, ulcerative colitis

CD patients in IBDQ remission (defined as IBDQ total score ≥170) had lower SF-7a values (mean 46.6) than patients with a severe (defined as IBDQ total score < 110) IBDQ score (mean 64.1, nominal P < 0.001; Fig. 1a). Similarly, UC patients in IBDQ remission had lower SF-7a values (mean 44.8) than patients with a severe IBDQ score (mean 64.3, nominal P < 0.001; Fig. 1b). Less fatigue (i.e., lower SF-7a mean scores) was associated with better general health and well-being (i.e., higher IBDQ mean scores). Of note, CD and UC patients in IBDQ remission had SF-7a fatigue mean T-scores < 50, indicating that these patients were less impacted by fatigue.

**Fig. 2:**
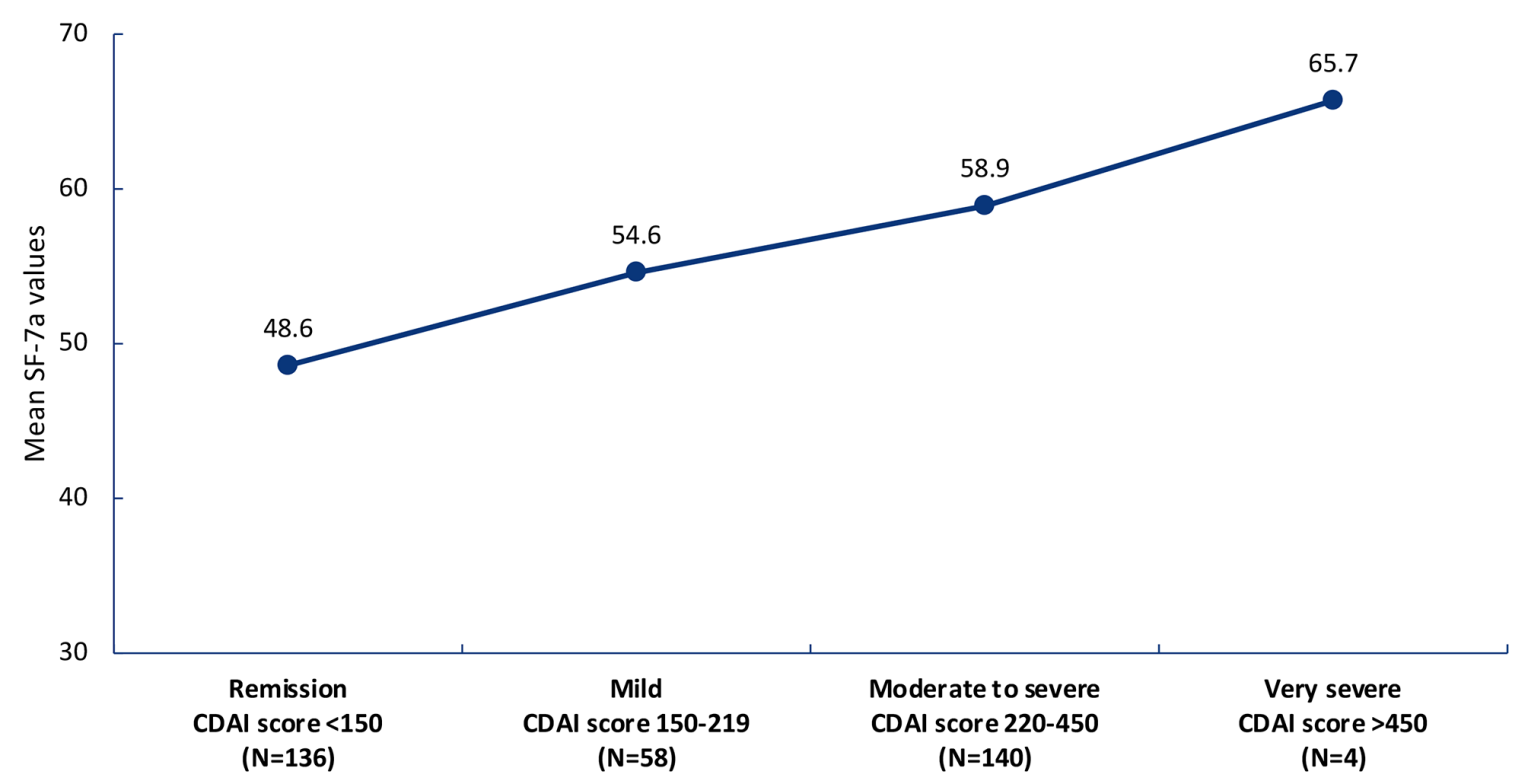
Mean PROMIS Fatigue SF-7a values, by disease severity groups by CDAI at Week 12 in patients with CD. *CD*, Crohn’s disease; *CDAI*, Crohn’s Disease Activity Index; *PROMIS*, Patient-Reported Outcomes Measurement Information System; *SF-7a*, Short Form-7a

CD patients in CDAI remission (defined as CDAI score < 150) had lower SF-7a values (mean 48.6) than patients with a very severe (defined as CDAI score > 450) CDAI score (mean 65.7, nominal P < 0.001; Fig. 2). Less fatigue (i.e., lower SF-7a scores) was associated with lower disease activity (i.e., lower CDAI scores). CDAI remission was associated with SF-7a fatigue mean T-scores < 50, indicating that patients in clinical remission were less impacted by fatigue.

**Fig. 3:**
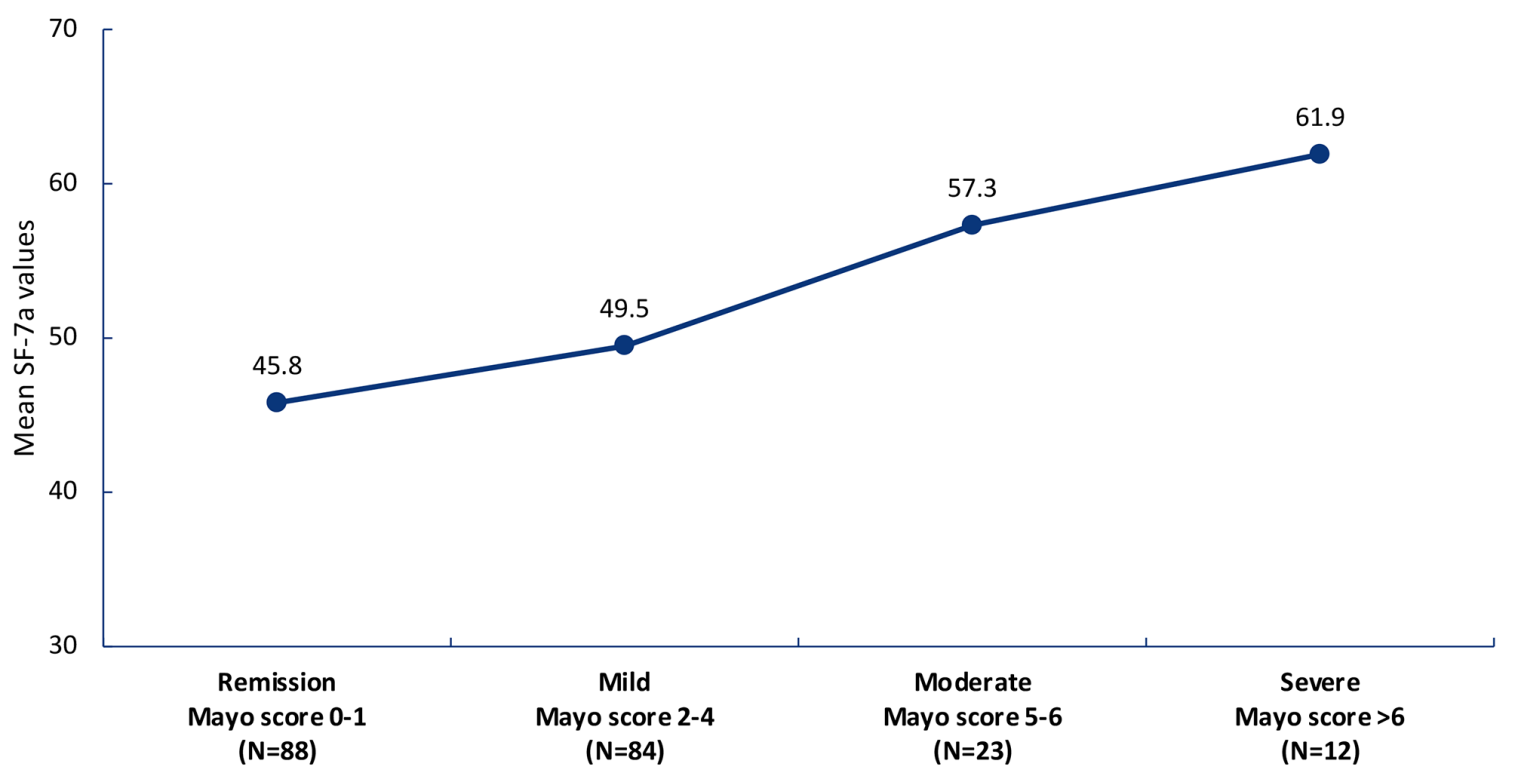
Mean PROMIS Fatigue SF-7a values, by disease severity groups by partial Mayo score at Week 12 in patients with UC. *PROMIS*, Patient-Reported Outcomes Measurement Information System; *SF-7a*, Short Form-7a; *UC*, ulcerative colitis

UC patients in remission (defined as partial Mayo score 0–1) had lower SF-7a values (mean 45.8) than patients with severe disease severity (mean 61.9, nominal P < 0.001; Fig. 3). UC patients who had moderately to severely active UC (defined as partial Mayo scores ≥5) had fatigue mean Tscores > 50, indicating that these patients were impacted by fatigue.

### Convergent and discriminant validity

Spearman correlations revealed that SF-7a mean scores were strongly correlated (r_s_ ≥ -0.70) with fatigue-related IBDQ items (e.g., “feeling of fatigue or tiredness”) in patients with CD and UC (Table 4). IBDQ item “energy levels” was also found to be correlated with SF-7a in CD.

(r_s_ = -0.77) and UC (r_s_ = -0.60) patients.

In contrast, relatively weaker correlations were observed with IBDQ items that were not directly fatigue-related, e.g., “accidental soiling of underpants” (CD: r_s_ = -0.44; UC: r_s_ = -0.50) and “rectal bleeding” (CD: r_s_ = -0.26; UC: r_s_ = -0.40). These findings further implied the convergent and divergent validity of the SF-7a scale.

**Table 4:** Convergent and discriminant analyses: Correlation between PROMIS Fatigue SF-7a scores and IBDQ items at Week 12

IBDQ questions/items	CD patients	UC patients
	(N = 305)	(N = 204)
Accidental soiling of underpants	-0.44	-0.50
Angry because of bowel problem	-0.54	-0.62
Avoid events where no washroom	-0.57	-0.61
Delay or cancel social engagement	-0.57	-0.60
Difficulty leisure/sports activity	-0.68	-0.62
Embarrassed by bowel problem	-0.59	-0.63
**Energy**	**-0.77**	-0.60
Fear of not finding a washroom	-0.54	-0.54
**Feeling of fatigue or tiredness**	**-0.80**	**-0.70**
Felt depressed or discouraged	-0.61	-0.68
Felt generally unwell	-0.65	-0.66
Felt irritable	-0.59	-0.63
Felt relaxed and free of tension	-0.61	-0.59
Felt tearful or upset	-0.52	-0.62
Frustrated, impatient, restless	-0.66	-0.66
Going to bathroom, empty bowels	-0.52	-0.54
Lack of understanding from others	-0.49	-0.62
Limited sexual activity	-0.50	-0.56
Loose bowel movements	-0.44	-0.50
Nausea, feeling sick to stomach	-0.48	-0.57
Problem large amounts of gas	-0.42	-0.53
Problem maintaining/losing weight	-0.51	-0.46
Problems with sleep	-0.62	-0.52
Rectal bleeding	-0.26	-0.40
Satisfied, happy with personal life	-0.53	-0.56
Troubled by abdominal bloating	-0.51	-0.52
Troubled by cramps in abdomen	-0.55	-0.56
Troubled by pain in the abdomen	-0.53	-0.50
Unable to attend school/do work	-0.57	-0.54
Worried or anxious	-0.55	-0.56
Worried, possibility of surgery	-0.45	-0.41

*CD*, Crohn’s disease; *IBDQ*, Inflammatory Bowel Disease Questionnaire; *PROMIS*, Patient-reported Outcomes Measurement Information System; *SF-7a*, Short Form-7a; *UC*, ulcerative colitis. Strong correlations (r_s_ ≥ -0.70) between fatigue-related IBDQ items and mean SF-7a scores are bolded

### Test-retest reliability

Test-retest reliability of the SF-7a scale was assessed among stable (defined as no change in PGIC from baseline to Week 12) CD and UC patients (Table 5). Mean SF-7a scores were similar at baseline and Week 12, with ICC values 0.54 in CD and 0.89 in UC. Overall, these findings indicate that the test-retest reliability of the SF-7a scale is adequate.

**Table 5:** Reliability of PROMIS Fatigue SF-7a scores among stable patients at baseline and Week 12

	No change in PGIC from baseline to Week 12 in patients with CD (N = 74)			No change in PGIC from baseline to Week 12 in patients with UC (N = 15)		
**PROMIS Scale**	**Baseline** **Mean (SD)**	**Week 12** **Mean (SD)**	**ICC** **(95% CI)**	**Baseline** **Mean (SD)**	**Week 12** **Mean (SD)**	**ICC** **(95% CI)**
Fatigue SF-7a	59.6 (7.0)	58.0 (7.9)	0.54 (0.38, 0.69)	62.4 (10.4)	63.2 (10.1)	0.89 (0.74, 0.96)

*CD*, Crohn’s disease; *CI*, confidence interval; *ICC*, intraclass correlation coefficient; *PGIC*, Patient Global Impression of Change; *PROMIS*, Patient-reported Outcomes Measurement Information System; *SD*, standard deviation; *SF-7a*, Short Form-7a; *UC*, ulcerative colitis

### Responsiveness and threshold for clinically meaningful change

The SF-7a scale was responsive to change in disease severity from baseline to Week 12 as assessed by the PGIC in both CD and UC patients (Table 6). Using the PGIC as an anchor variable, CD and UC patients with greater improvements in PGIC had greater reductions in SF-7a scores. A 3-level change (i.e., improvement) of feeling “a lot better” was associated with an 11.5-point mean reduction in the SF-7a scale in CD patients and an 11.1-point mean reduction in UC patients. Similarly, a 2-level change (i.e., improvement) of feeling “moderately better” was associated with a 6-point mean reduction in the SF-7a scale in CD patients and a 6.4-point mean reduction in UC patients.

**Table 6:** Responsiveness of PROMIS Fatigue SF-7a scale: Change from baseline in fatigue score at Week 12

PGIC from baseline to Week 12	CD					UC				
	N	Mean (SD)	SES	SRM	Guyatt’s	N	Mean (SD)	SES	SRM	Guyatt’s
A lot better	71	-11.5 (8.5)	-1.6	-1.4	-1.6	106	-11.1 (8.4)	-1.3	-1.3	-1.1
Moderately better	63	-6.0 (9.1)	-0.7	-0.7	-0.9	43	-6.4 (8.0)	-0.9	-0.8	-0.6
A little better	94	-4.3 (6.9)	-0.5	-0.6	-0.6	34	-5.7 (8.1)	-0.6	-0.7	-0.6
No change	74	-1.6 (7.0)	-0.2	-0.2	-0.2	15	0.9 (4.7)	0.1	0.2	0.1
A little worse to a lot worse	15	-2.4 (11.1)	-0.3	-0.2	-0.3	6	-0.1 (7.9)	-0.03	-0.01	-0.01

*CD*, Crohn’s disease; *Guyatt’s*, Guyatt’s responsiveness statistic; *PGIC*, Patient Global Impression of Change; *PROMIS*, Patient-Reported Outcomes Measurement Information System; *SD*, standard deviation; *SES*, standardized effect size; *SF-7a*, Short Form-7a; *SRM*, standardized response mean; *UC*, ulcerative colitis

## Discussion

This study evaluated the content validity and psychometric properties of the PROMIS-Fatigue SF-7a v1.0 scale to determine its suitability in clinical trials to assess fatigue in patients with moderately to severely active CD and UC. Our analysis demonstrated that all patients with moderately to severely active CD and UC reported fatigue as a common symptom. Patients who participated in the qualitative interview confirmed that SF-7a items were relevant to assessing fatigue, the instructions and response options were clear, and its 7-day recall period was appropriate. In addition, higher SF-7a scores were associated with higher disease activity (CDAI and Mayo score) and lower HRQoL (IBDQ), confirming known groups validity. The correlation of the SF-7a scale was higher with fatigue-related items (r_s_ ≥ -0.70) than with items not directly associated with fatigue. Test-retest reliability was moderate to good (0.54 to 0.89) among patients with stable disease, and responsiveness to change in disease severity was demonstrated from baseline to Week 12 (e.g., “moderately better” means ~ 6-point improvement). A ≥7-point decrease was identified as a reasonable threshold to define clinically meaningful improvement.

Fatigue in chronic diseases has been clinically described as a “persistent, overwhelming sense of tiredness, weakness, or exhaustion resulting in a decreased capacity for physical and/or mental work,” and is typically unrelieved by adequate sleep or rest [25]. Furthermore, fatigue can exist as a unique entity and not merely a component of psychological comorbidity or “illness behavior” [25]. The prevalence of fatigue in moderately to severely active CD and UC patients supports the rationale to have an IBD-specific PRO instrument to assess fatigue in clinical trials.

There are numerous PRO instruments that have been used to measure fatigue in IBD clinical studies. Common fatigue instruments, such as the Multidimensional Fatigue Symptom Inventory-Short Form (MFSI-SF), Brief Fatigue Inventory (BFI), and Functional Assessment of Chronic Illness Therapy-Fatigue (FACIT-F), have all been reported to be valid and reliable in assessing fatigue in other disease conditions, but their application remains limited in patients with IBD [26].

MFSI-SF, a 30-item instrument, measures fatigue in 5 multidimensional subscales over the previous week, and appears to be sensitive in detecting change in cancer-related fatigue [27]. BFI assesses fatigue severity and interference with daily function over the past 24 h [28]; however, its test-retest reliability after repeated administrations remains a concern [29]. FACIT-F, a 13-item instrument originally designed to assess cancer-related anemia [30], has been validated in the general population as well as in patients with a number of other disease conditions. However, some items included in the FACIT-F may be interpreted as not relevant to fatigue [31], raising concerns on content validity. During a recent cognitive debriefing of FACIT-Fatigue, CD (N = 30) and UC (N = 33) patients were asked to report whether anything relevant to their fatigue experience was missing from the FACIT-Fatigue questionnaire [32]. Mental health/mental fatigue that can lead to changes in one’s thinking process or mood was reported as a missing concept by 6 patients with CD and 1 patient with UC. Mental fatigue is assessed by one item in PROMIS-Fatigue SF-7a (“How often were you too tired to think clearly”?).

This study assessed the content validity and psychometric properties of the SF-7a scale in patients with IBD. In the qualitative analysis, all CD and UC patients identified fatigue as the most common symptom, with all patients having spontaneously reported experiencing fatigue. Patients with CD or UC often talked about their fatigue intensity/severity and fatigue frequency together using expressions such as “constantly exhausted,” or “wiped out all the time”, indicating both fatigue frequency and intensity are important attributes in the evaluation of fatigue experience.

Cognitive debriefing of the SF-7a scale confirmed the content validity of the SF-7a scale and showed that most patients correctly interpreted each item, felt each item was relevant to their experience, and found the questionnaire was easy to recall and complete. The SF-7a scale was designed to assess fatigue severity from general tiredness and lack of energy to exhaustion and mental tiredness with a response option on a 5-point Likert scale to capture frequency over the past 7 days. Assessing both fatigue intensity and frequency using only 7 items may make the SF-7a scale more valuable in evaluating fatigue in the IBD population.

We have also demonstrated that the SF-7a scale was reliable, valid, and responsive to change in patients with moderately to severely active CD and UC. The SF-7a scale was sensitive in distinguishing clinically different groups as defined by IBDQ, CDAI, and partial Mayo score. The SF-7a scale had adequate reliabilities, and as expected, the strongest correlations (r_s_ ≥ -0.70) were identified with fatigue-related IBDQ items versus relatively weaker correlations with other IBDQ items not directly assessing fatigue. Compared to the weak correlation of FACIT-F with clinical outcomes in CD patients compared with UC patients as described in a previous study [26], the current psychometric analysis revealed that SF-7a demonstrated similar correlations with clinical outcomes, regardless of UC or CD disease indication. These findings contribute to the supportive clinical evidence for the use of the PROMIS-Fatigue SF-7a scale in clinical trials for patients with CD and UC.

While the SF-7a scale has been accepted by the Food and Drug Administration to evaluate the treatment effect on fatigue in patients with myelofibrosis [33], the qualitative and quantitative analysis presented herein may provide evidence supporting SF-7a scale as a valid instrument to evaluate fatigue for disease state and progression for patients with CD and UC. One strength of our analysis was that the patients enrolled in both qualitative and quantitative studies had similar disease severity at baseline (moderately to severely active). Also, anchor-based analyses were used to assess changes in clinical outcomes (e.g., HRQoL, disease activity, and disease severity) as they relate to mean SF-7a fatigue values, both of which meet the general requirements for developing a PRO instrument for inclusion in clinical trials that are fit-for-purpose and meaningful to patients, clinicians, regulators, and payers [34]. Moreover, it was identified that a 6- to 6.4-point reduction in the SF-7a scale was clinically meaningful, which may help facilitate informed decisions in establishing appropriate response criteria for detecting clinically meaningful thresholds with the SF-7a scale. Since two levels of improvement in PGIC was generally considered a clinically meaningful change in disease severity from a patient perspective, the observed change in SF-7a among those patients was defined as meaningful change thresholds. Therefore, a ≥7-point change is a preliminary estimate and can be used as a conservative cutoff to define fatigue response in both CD and UC patients.

A potential limitation of this study is that the Phase 2 clinical trials which provided the data for evaluation of the measurement properties enrolled a relatively homogenous patient population, since all patients were required to have moderately to severely active disease. Fatigue assessment by SF-7a may be limited among patients with moderately to severely active disease, additional evaluation of fatigue using SF-7a in patients with mild disease is needed. In addition, due to the limited sample size, there were no suitable analyses to determine whether the SF-7a scale was responsive to worsening of disease. Other limitations include the patient population in the qualitative study being restricted to US patients, the time period in which the reliability assessments were conducted, and the lack of specific hypotheses for correlations between overall disease severity and systemic fatigue. Shorter time intervals between test-retest may prove to yield improved reliabilities, particularly in CD patients. Further validation studies of IBD patients from real-world settings are needed and responsiveness to efficacious treatment vs. placebo needs to be further tested.

In conclusion, we have demonstrated that the SF-7a scale is a valid, reliable, and sensitive measure of fatigue in patients with moderately to severely active CD or UC and can be used to evaluate treatment response. These findings provide supportive evidence for the use of the SF-7a scale in clinical studies of patients with moderately to severely active CD and UC. These data may be helpful in informing future clinical trials as clinicians seek to understand how potential treatment options in IBD may impact fatigue.

## Data Availability

The request to share data underlying this article will be individually assessed upon reasonable request to the corresponding author.

## Abbreviations

BFI: Brief Fatigue Inventory
CD: Crohn’s disease
CDAI: Crohn’s Disease Activity Index
FACIT-F: Functional Assessment of Chronic Illness Therapy-Fatigue
HRQoL: health-related quality of life
IBD: inflammatory bowel disease
IBDQ: Inflammatory Bowel Disease Questionnaire
ICC: intraclass correlation coefficients
MFSI-SF: Multidimensional Fatigue Symptom Inventory-Short Form
PGIC: Patient Global Impression of Change
PRO: Patient-Reported Outcome
PROMIS: Patient-Reported Outcomes Measurement Information System®
SD: standard deviation
SES: standardized effect size
SF-7a: Short Form-7a
SRM: standardized response mean
UC: ulcerative colitis
US: United States
